# Autistic People’s Perinatal Experiences I: A Survey of Pregnancy Experiences

**DOI:** 10.1007/s10803-022-05754-1

**Published:** 2022-10-19

**Authors:** Sarah Hampton, Carrie Allison, Simon Baron-Cohen, Rosemary Holt

**Affiliations:** Autism Research CentreDepartment of Psychiatry, University of Cambridge, Cambridge, UK

**Keywords:** autism, pregnancy, healthcare, motherhood

## Abstract

Qualitative studies of autistic people’s pregnancy experiences have indicated sensory and communication related barriers to accessing adequate prenatal healthcare. However, quantitative work on the topic is scarce. This online survey study explored pregnancy experiences among 417 autistic and 524 non-autistic people. Compared with non-autistic people, autistic people reported heightened sensory and physical experiences during pregnancy and were more likely to experience prenatal depression and anxiety. Autistic people experienced lower satisfaction with prenatal healthcare, including having lower perceptions of their relationships with healthcare professionals and greater difficulties with antenatal classes. This study identifies key adjustments that can be made to prenatal healthcare, including sensory and communication adjustments. The findings highlight the need for greater autism understanding and awareness among professionals.

## Introduction

Research exploring the pregnancy experiences of autistic people is scarce. There is a growing body of literature, however, assessing the pregnancy experiences of those with disabilities, including those with mental health challenges and those with intellectual disability. These experiences could inform understanding of those of autistic people given that intellectual disability and mental health conditions often co-occur with autism and may bring similar challenges.

Secondary analyses of UK national survey data have revealed gaps in care for disabled people during pregnancy. One study analysed survey data from 2015 relating to the maternity experiences of disabled and non-disabled people (Malouf et al., 2017). They found that disabled people (including those with physical disabilities, mental health conditions, sensory disabilities and intellectual disabilities) had lower perceptions of pregnancy care including being less likely than people without disabilities to report being spoken to by health professionals in a way they could understand and being less likely to report being involved in decisions about their care. Additionally, people with physical and mental health disabilities were less likely than people without disabilities to report having time to ask questions in prenatal appointments and less likely to feel listened to by professionals during pregnancy.

These results echo those of a previous analysis of national survey data from 2010, which also found lower perceptions of perinatal care among people with disabilities and additionally indicated that disabled people were less likely than non-disabled people to attend antenatal classes (Redshaw et al., 2013). A further survey study focusing on people with mental health conditions found that they perceived maternity care less positively than those without mental health conditions (Henderson et al., 2018). This included being less likely to feel that doctors talked to them in a way they could understand, treated them respectfully and listened to them. A small study of disabled people’s experiences in the UK and Ireland found that the majority of those surveyed felt that reasonable adjustments to maternity care had not been made for them and that maternity care professionals did not have appropriate awareness of disability (Hall et al., 2018).

These first-hand perceptions of poorer healthcare among disabled people have been supported by research exploring healthcare professionals’ perspectives on delivering care to those with disabilities. Research examining midwives’ confidence in caring for people with disabilities has revealed that midwives do not feel they have sufficient education and information to adequately care for those with mental health conditions (Noonan et al., 2018) nor intellectual disability (Homeyard et al., 2016). In addition, a UK survey revealed that the majority of NHS trusts did not have routine antenatal information adapted for people with intellectual disability nor routinely offered extra time in appointments to people with intellectual disability (Homeyard & Patelarou, 2018).

Few studies have explored pregnancy among autistic people. Studies taking a qualitative approach have tended to report issues surrounding sensory experiences during pregnancy and difficulties interacting with professionals. A case study of one autistic woman reported that she experienced heightened sensory sensitivities during pregnancy and encountered difficulties communicating with maternity healthcare professionals who she felt did not respect her wishes and had little understanding of how autistic people experience pregnancy (Rogers et al., 2017). Another study explored the perinatal experiences of eight autistic women (Gardner et al., 2016). The women reported enhanced sensory sensitivities to bright lights, sound, smell and touch during pregnancy, and sensory sensitivities sometimes made certain aspects of prenatal appointments challenging, such as bright lights and touch. The mothers did not always disclose their autism diagnosis to professionals and reported that they required direct and clear information when interacting with professionals. Heightened sensory experiences during pregnancy, including difficulties with the sensory aspects of medical appointments, were also found in another study of 7 autistic women’s experiences (Talcer et al., 2021).

The communication and sensory issues identified in these studies fit with findings from the broader literature surrounding access to healthcare for autistic people. Autistic people can face sensory-related barriers to healthcare such as difficulties with the sensory environment of healthcare facilities (Raymaker et al., 2017), in addition to communication barriers such as difficulty processing verbal information (Raymaker et al., 2017) and a lack of accessible communication formats such as written information (Nicolaidis et al., 2015). Furthermore, autistic people can feel that healthcare professionals lack adequate knowledge about autism and that this lack of knowledge can be a barrier to receiving appropriate healthcare (Nicolaidis et al., 2015).

There are few quantitative studies exploring pregnancy among autistic people. Using Swedish national medical data from 2006 to 2014, one study compared the pregnancy and birth outcomes of 2198 autistic people and 877,742 non-autistic people, covarying for the mother’s age, country of birth, smoking, BMI, parity, psychotropic and antiepileptic medication during pregnancy and the year of giving birth (Sundelin et al., 2018). They found that autistic people had an increased risk of preeclampsia, which the authors speculated may be due to altered immune response in autistic people. Autistic people were not found to be at increased risk of gestational diabetes. Pohl et al. surveyed 355 autistic mothers and 132 non-autistic mothers about their experiences of pregnancy, birth and motherhood (Pohl et al., 2020). Regarding pregnancy experiences, autistic mothers were more likely to experience prenatal depression than non-autistic mothers, though were just as likely to attend antenatal classes as non-autistic mothers. Autistic mothers were also more likely to report difficulties in communicating with professionals (e.g. teachers, clinicians, social workers) about their child, more likely to experience so much anxiety that it affects their ability to communicate when interacting with professionals and more likely to feel misunderstood by professionals. Autistic mothers were reluctant to disclose their autism diagnosis to professionals and worried that professionals’ attitude towards them would change if they disclosed.

There is currently no quantitative research focusing primarily on the pregnancy experiences of autistic people. This study aimed to explore perceptions of prenatal healthcare among autistic people, in order to identify gaps in current practice, as well as exploring autistic people’s physical and sensory experiences during pregnancy.

## Methods

### The Survey

The survey contained three sections: pregnancy, childbirth and postnatal experiences. This paper reports on the pregnancy section, while the other sections are reported on elsewhere (Hampton et al., 2022). The pregnancy section covered: (1) sensory and physical experiences during pregnancy; (2) experiences of prenatal appointments; and (3) support during pregnancy. The survey questions can be found in Online Resource 1.

The survey contained forced choice and open-ended questions. The forced choice questions most often required one of: ‘strongly agree’, ‘somewhat agree’, ‘somewhat disagree’, ‘strongly disagree’, ‘don’t know’ or ‘not applicable’. Some questions were presented depending on the response given to a previous question. For example, participants were only asked whether they had found it helpful to have an advocate during prenatal appointments if they had previously indicated that they had an advocate. Questions concerning autism were only asked to those in the autistic group. The survey also contained demographic questions and the 10-item version of the Autism Quotient (AQ-10) (Allison et al., 2012), a self-report measure of autistic traits. Scores on the AQ-10 range from 0 to 10, with a score of six or above indicating that a clinical assessment for autism may be warranted.

The findings from a separate qualitative study exploring autistic women’s perinatal experiences (Hampton et al., 2022) were used as a foundation for choosing the topics covered. Additionally, feedback from the autistic community was sought through Twitter. Comments on which aspects of pregnancy autistic followers would like to see more research on were taken into account when creating the survey. Three autistic mothers gave feedback on the phrasing and content of the survey questions via email. Each of the mothers worked with other autistic mothers in a professional capacity, one as a midwife, another as a doula and another as a researcher.

Participants completed the survey online and indicated their informed consent electronically. Responses were anonymous and ethical approval was obtained from the University of xxxxxx Ethics Committee, PRE.2018.093.

### Participants

Participants were recruited through the Cambridge Autism Research Database (CARD), parenting groups, autism support groups and social media. Participants were eligible if they were at least 18 years old and had either given birth at least once or if they had never given birth but were currently in the third trimester of pregnancy. Those in the first or second trimester were not eligible as many questions were less relevant to those in the early stages of pregnancy, and these participants were not asked to reflect on a previous pregnancy, as their experience of their current pregnancy may have affected their recollection. For those not currently pregnant, participants were asked to reflect on their most recent pregnancy that went to term.

In total, 245 people with a diagnosis of autism, 172 people who believed themselves to be autistic but did not have a formal diagnosis and 524 non-autistic people (who neither had a diagnosis nor believed themselves to be autistic) were included in the study. Post-hoc sensitivity power analysis indicated that for the total sample (n = 941), there was adequate (80%) power to detect small effect sizes (odds ratio ≥ 1.70), with a two-tailed alpha of 0.05.

Those who believed themselves to be autistic but did not have a diagnosis were included in the autistic group. This is because the mean AQ-10 score of the self-identifying group was above the cut-off of six (mean = 7.02, SD = 2.10), their AQ-10 mean score was not significantly different from that of those with a diagnosis (mean = 7.87, SD = 1.66, p = 0.28) and they scored significantly higher than the non-autistic group (mean = 1.96, SD = 1.64, p < 0.001). This approach follows that of a previous similar paper (Pohl et al., 2020). Many autistic adults may have not had the opportunity to receive a diagnosis earlier in life due to barriers such as changes to diagnostic criteria over time, lack of access to diagnostic services, waiting lists, costs and anxiety around appointments (Lai & Baron-Cohen, 2015; Lewis, 2017). Barriers may be pronounced for those, such as women, who may present atypically and engage in greater masking of their autistic characteristics (Lockwood Estrin et al., 2021). As such it is important that research captures the experiences of those autistic individuals who have not received a diagnosis. There is some evidence that self-referrals to adult autism diagnostic services are more likely to result in an autism diagnosis than other referrals, and that self-referral may be a particularly effective route to diagnosis for women (Whitney & Stansfield, 2019), supporting the accuracy of self-identification. As experiences of prenatal healthcare may differ depending on whether or not one has an autism diagnosis, group comparisons on the healthcare related survey questions were conducted between participants with a diagnosis of autism and participants who self-identified as autistic but did not have a diagnosis. Results of these analyses are presented in Online Resource 2 and reveal minimal group differences, supporting the inclusion of self-identifying participants within the autistic group.

The autistic and non-autistic groups did not differ significantly on education, ethnicity, whether their most recent pregnancy was singleton or multiple, or on total number of pregnancies or live births (Table 1). The groups significantly differed on country of residence, current age, age at most recent birth and current partner status. The autistic group were significantly more likely to identify as non-binary/other gender, had significantly lower annual household income, were significantly more likely to have ever been diagnosed with a psychiatric condition and gave birth to their youngest child significantly longer ago than the non-autistic group.

**Table 1:** Demographic information for the autistic and non-autistic groups

	Non-autistic group	Autistic group	p-value
**Mother’s current age** ^**a**^			**0.046**
N	524	417	
Mean (SD)	41.68 (10.10)	42.94 (9.17)	
**Mother’s age at most recent birth** ^**a**^			**0.002**
N	524	417	
Mean (SD)	33.23 (5.09)	32.14 (5.31)	
**Gender identity** ^**b**^			**< 0.001**
N	524	417	
Female	523 (100%)	384 (92%)	
Male	0 (0%)	1 (0.24%)	
Non-binary/Other	1 (0.19%)	32 (8%)	
**Education** ^**b**^			0.22
N	524	417	
Completed high school	95 (18%)	93 (22%)	
Undergraduate degree	218 (42%)	156 (37%)	
Postgraduate degree	187 (36%)	142 (34%)	
Other	24 (5%)	26 (6%)	
**Income** ^**b**^			**< 0.001**
N	513	410	
Greater than £100,000	88 (17%)	44 (11%)	
£50,000-£100,000	177 (35%)	95 (23%)	
£25,000-£50,000	162 (32%)	139 (34%)	
Less than £25,000	86 (17%)	132 (32%)	
**Current partner status** ^**b**^			**< 0.001**
N	524	417	
Married/in a partnership	457 (87%)	313 (75%)	
Divorced/separated/widowed	36 (7%)	62 (15%)	
Single	31 (6%)	41 (10%)	
**Country** ^**b**^			**< 0.001**
N	524	417	
UK	364 (70%)	249 (59%)	
USA	52 (10%)	82 (20%)	
Ireland	57 (11%)	12 (3%)	
Other	51 (10%)	74 (18%)	
**Ethnicity** ^**b**^			0.38
N	521	412	
White	490 (94%)	393 (95%)	
Non-white	31 (6%)	19 (5%)	
*Asian*	9 (2%)	1 (0.24%)	
*Black African/Black* *Caribbean*	1 (0.02%)	0 (0%)	
*Mixed ethnicity*	14 (3%)	7 (2%)	
*Other*	7 (1%)	11 (3%)	
**Psychiatric condition(s)** ^**b**^			**< 0.001**
N	523	411	
Yes	190 (36%)	273 (66%)	
No	333 (64%)	138 (34%)	
**AQ-10 score** ^**c**^			**< 0.001**
N	524	417	
Mean (SD)	1.96 (1.64)	7.52 (1.90)	
**Total number of pregnancies** ^**c**^			0.39
N	524	417	
Mean (SD)	2.88 (1.78)	3.06 (2.00)	
**Total number of live births** ^**c**^			0.17
N	524	417	
Mean (SD)	1.97 (1.05)	2.09 (1.13)	
**Age of youngest child in years** ^**c**^			**< 0.001**
N	524	417	
Mean (SD)	8.45 (8.58)	10.80 (8.88)	
**Singleton or multiple birth (youngest child)** ^**b**^			0.93
N	524	417	
Singleton	511 (98%)	407 (98%)	
Multiple	13 (2%)	10 (2%)	

*Note*. SD = standard deviation

^a^T-test performed

^b^Fisher’s exact test performed

^c^Wilcoxon rank-sum test performed

p-values in bold are significant at p < 0.05

### Data Analysis

Ineligible participants were excluded, including those under 18 years old (1 participant) and those who had never given birth nor were in the third trimester of pregnancy (13 participants). Participants were excluded if they were suspected to be duplicates, that is, if they had the same anonymous participant code as another participant and gave the same demographic responses (30 participants). Duplicates may have arisen due to participants re-starting the survey after the initial link expired. Anyone who had not answered at least 20% of the survey questions beyond the demographic questions was excluded (197 participants).

‘Strongly agree’ and ‘somewhat agree’ responses were combined into an ‘agree’ category and ‘strongly disagree’ and ‘somewhat disagree’ were combined into a ‘disagree’ category, in order to facilitate analysis with logistic regression. Similarly, ‘very satisfied’ and ‘somewhat satisfied’ were reduced to ‘satisfied’, and ‘very dissatisfied’ and ‘somewhat dissatisfied’ were reduced to ‘dissatisfied’. ‘Don’t know’ and ‘Not applicable’ responses were excluded from analysis.

Where possible, thematically similar items were analysed in a multivariate manner in order to account for correlations among items. This was achieved by reshaping the data into long format such that responses for all items were aggregated into one binary (agree/disagree) outcome variable. In this manner, items were effectively treated as repeated measures. A multilevel binary logistic regression was then performed with the agree/disagree response variable as the outcome and group as a predictor. Each model included a random intercept for participant to account for dependency due to repeated measures. A group by item interaction term was included in each model in order to obtain odds ratios and confidence intervals for each individual item. Items that correlated negatively with the other items within the multivariate analysis were reverse scored prior to analysis. To obtain an omnibus analysis of the effect of group across the items as a whole, a likelihood ratio test was performed comparing the model with group as a predictor and the model without group as a predictor; if the model with group as a predictor was a significantly better model than that without, group was considered to have a significant effect on responses across the items as a whole. Only if this omnibus test was significant were analyses relating to individual items presented.

Decisions to group items together in a multivariate analysis were based on thematic similarity between items (e.g. questions regarding prenatal appointments were analysed together, questions regarding senses were analysed together etc.). Polychoric correlations between theoretically related items were also conducted (see Online Resource 3). Thematically similar items were generally at least moderately correlated (r ≥ 0.30 was considered moderate (Cohen, 1992)), supporting a multivariate analysis. Some items were only weakly correlated with others and were excluded from the multivariate analysis. For example, items concerning attending appointments were strongly correlated with each other though had few correlations of r ≥ 0.30 with other prenatal healthcare items, and therefore were analysed together in a multivariate analysis but excluded from the main prenatal healthcare multivariate analysis.

Some items that were survey logic dependent (only presented depending on the response to a prior question) were excluded from multivariate analyses. For example, the item, ‘I found it helpful to have an advocate during prenatal appointments’ was asked only if participants previously indicated having an advocate and the item, ‘I would have found it helpful to have an advocate during prenatal appointments’ was asked if participants indicated not having an advocate. These questions were therefore not entered together into a multivariate analysis and were analysed individually.

Correction for multiple comparisons was not applied to analyses of individual items within a multivariate analysis, though all other analyses were FDR corrected (i.e. all analyses of individual items not included within a multivariate analysis and omnibus tests of the overall effect on group on multiple items were corrected for together). P-values of less than 0.05 are considered significant.

All analyses included the following covariates: mothers’ age at giving birth, time passed since giving birth (age in days of their youngest biological child), the number of live births the participant had experienced, and country of residence. The adjusted odds ratio (aOR) is reported for each analysis. Those participants with missing data for any covariate were excluded from analyses (12 participants). While current partner status, income and the presence of psychiatric conditions significantly differed between the two groups these were not included as covariates. Current partner status may not reflect partner status at the time being reported on and as such may be less influential than other factors. Missing data was greater for income than other covariates and as such including income would have resulted in a reduced sample size. Finally, as psychiatric conditions commonly co-occur with autism (Lai et al., 2019) and factors surrounding autism may in fact contribute to the development of psychiatric conditions (Cage et al., 2018), attempting to disentangle autism from these other conditions may lead to a significant aspect of the autistic experience being obscured.

While the quantitative data are the focus of this paper, quotes from responses to open-text questions are sometimes reported in order to elucidate the quantitative data. A full qualitative analysis (Braun & Clarke, 2006) was not conducted and as such, the open-text response data are intended to provide preliminary, speculative elucidation of the quantitative findings.

## Results

### Sensory and Physical Experiences During Pregnancy

**Sensory experiences**.

Participants were asked whether each of their senses were heightened, reduced or stayed the same when pregnant compared to when not pregnant. A multivariate multinomial logistic regression was performed across the five senses as a whole. A model including group as a predictor was a significantly better fit than the model without group, *X*^2^(10) = 251.64, p < 0.001, indicating that there was a significant effect of group across the senses as a whole. For each sense, the autistic group was more likely than the non-autistic group to report that the sense had been heightened (as opposed to no change) during pregnancy (Table 2). There were no significant group differences in reporting a reduction in sensation (as opposed to no change) for smell, taste or vision. The autistic group was more likely than the non-autistic group to report a reduction in touch and hearing during pregnancy.

**Table 2:** Sensory changes during pregnancy

	Non-autistic group	Autistic group	aOR (95% CI)	p-value
Smell				
N	523	413		
Heightened	401 (77%)	344 (83%)	2.28 (1.12–4.66)	**0.02**
Stayed the same	120 (23%)	64 (16%)	-	-
Reduced	2 (0.38%)	5 (1%)	4.51 (0.68–30.03)	0.12
Taste				
N	524	413		
Heightened	281 (54%)	288 (70%)	3.78 (1.95 - 7.24)	**< 0.001**
Stayed the same	226 (43%)	114 (28%)	-	-
Reduced	17 (3%)	11 (3%)	1.18 (0.41–3.42)	0.76
Touch				
N	522	413		
Heightened	119 (23%)	217 (53%)	9.43 (4.89 - 18.18)	**< 0.001**
Stayed the same	399 (76%)	188 (46%)	-	-
Reduced	4 (1%)	8 (2%)	4.27 (1.04–17.63)	**0.045**
Hearing				
N	521	411		
Heightened	47 (9%)	144 (35%)	11.31 (6.36–27.06)	**< 0.001**
Stayed the same	463 (89%)	248 (60%)	-	-
Reduced	11 (2%)	19 (5%)	3.54 (1.28–9.76)	**0.02**
Vision				
N	521	409		
Heightened	20 (4%)	66 (16%)	6.12 (2.52–15.06)	**< 0.001**
Stayed the same	440 (84%)	293 (72%)	-	-
Reduced	61 (12%)	50 (12%)	1.22 (0.61–2.42)	0.58

*Notes*. aOR = adjusted odds ratio; CI = confidence intervals

[Table 2 here]

Participants were asked how frequently they became overwhelmed by each sense when pregnant. For each sense, a score from 0 to 8 was allocated (‘Never’ = 0, ‘Several times a day’ = 8, intermediate response categories are detailed in Online Resource 4, Supplementary Table 1). A multivariate negative binomial regression was performed across the five senses as a whole. Negative binomial analysis was considered appropriate due to the right skewed nature of the data and the variance of the data being larger than the mean. A model including group as a predictor was a better fit than the model without group, *X*^2^(5) = 434.38, p < 0.001, indicating that there was a significant effect of group across the five senses as a whole. Participants were also asked how frequently they were overwhelmed by each sense when not pregnant. The frequency of being overwhelmed by the senses when not pregnant was included as a covariate so as to account for baseline differences in sensory experiences between the groups. For each sense, the autistic group were overwhelmed significantly more frequently than the non-autistic group.

### Bodily Changes During Pregnancy: Interoception and Proprioception

A multivariate binary logistic regression was performed for the items concerning interoception (awareness of one’s internal bodily sensations) and proprioception (awareness of the position and movement of the body). A model including group as a predictor was a better fit than the model without group, *X*^2^(2) = 98.22, p < 0.001, indicating a significant group difference. The autistic group were significantly more likely than the non-autistic group to report changes in their interoception (69% vs. 42% reported a change) and proprioception (38% vs. 15%; Table 3). Logistic regression revealed that the autistic group were significantly more likely to report difficulty adjusting to bodily changes associated with pregnancy (54% vs. 31%).

**Table 3:** Interoception, proprioception and bodily changes during pregnancy

	Non-autistic group	Autistic group	aOR (95% CI)	p-value	p-value (FDR adjusted)
Did you notice any changes since becoming pregnant in your interoception?			3.87 (2.69–5.56)	**< 0.001**	**-**
N	523	417			
Yes	218 (42%)	286 (69%)			
No	305 (58%)	131 (31%)			
Did you notice any changes since becoming pregnant in your proprioception?			3.97 (2.70–5.85)	**< 0.001**	**-**
N	523	416			
Yes	81 (15%)	158 (38%)			
No	442 (85%)	258 (62%)			
I found it very difficult to adjust to the changes my body went though^a^			2.87 (2.15–3.84)	**< 0.001**	**< 0.001**
N	524	413			
Agree	165 (31%)	223 (54%)			
Disagree	335 (64%)	167 (40%)			
Don’t know	5 (1%)	10 (2%)			
Not applicable	19 (4%)	13 (3%)			

*Notes.* Multivariate binary logistic regression performed; FDR = false discovery rate

^a^Item analysed with individual logistic regression due to weakly correlating with other items

When responding to open text questions asking them to describe their interoception and proprioception changes, participants in both groups reported a diversity of experiences, including experiencing bodily sensations more acutely, ‘*incredibly intense and overwhelming. I felt everything inside my body*’ (autistic participant), and feeling these sensations less clearly, ‘*I was somewhat disconnected from my body and was less able to recognize how I felt*’ (autistic participant).

[Table 3 here]

### Nausea During Pregnancy

The autistic group were significantly more likely to report experiencing more frequent nausea, with over half (51%) of this group reporting experiencing nausea all day every day (Table 4). Participants were asked to report the frequency of their nausea only for the time of their pregnancy when they were experiencing nausea (for example, someone who experienced nausea throughout the first trimester would be reporting on the first trimester only).

**Table 4:** Frequency of nausea during pregnancy

	Non-autistic group	Autistic group	aOR (95% CI)	p-value	p-value (adjusted)
Nausea			1.65 (1.28–2.14)	**< 0.001**	**< 0.001**
N	506	386			
Nausea every day and it lasted throughout the day	196 (39%)	197 (51%)			
Nausea every day and it did not last throughout the day	119 (24%)	74 (19%)			
Nausea less frequently than every day	115 (23%)	72 (19%)			
No nausea during the pregnancy	76 (15%)	43 (11%)			

*Note.* Ordinal logistic regression performed

[Table 4 here]

### Meltdowns and Shutdowns During Pregnancy

Scores from 0 (never) to 8 (several times a day) were allocated for the frequency of experiencing meltdowns (defined as becoming completely overwhelmed by the current situation and expressing this verbally (e.g. shouting, screaming, crying) or physically (e.g. kicking, lashing out, biting)), and shutdowns (defined as withdrawing from the world around oneself, for example being unable to communicate, lying down and being completely still and not being able to move). Questions concerning the frequency of meltdowns and shutdowns during pregnancy were explored with a multivariate negative binomial regression. A model including group as a predictor was a better fit than the model without group, *X*^2^(2) = 131.32, p < 0.001. The frequency of experiencing meltdowns and shutdowns when not pregnant was included as a covariate to account for baseline differences between the two groups. The autistic group were significantly more likely than the non-autistic group to report a higher frequency of experiencing meltdowns and shutdowns during pregnancy, with approximately one third of the autistic group indicating that meltdowns and shutdowns occurred twice a week or more (Online Resource 4, Supplementary Table 2). Questions concerning whether meltdowns and shutdowns were more intense during pregnancy than when not pregnant were analysed with multivariate binary logistic regression. A model including group as a predictor was a better fit than the model without group, *X*^2^(2) = 7.84, p = 0.03. The groups did not significantly differ in the tendency to report that the meltdowns experienced during pregnancy were more intense than those experienced when not pregnant, though the autistic group were more likely to indicate that shutdowns experienced during pregnancy were more intense than when not pregnant. In both groups, more participants agreed that meltdowns and shutdowns were more intense during pregnancy than disagreed.

### Pregnancy Conditions

The autistic group were significantly more likely to have pelvic girdle pain and vaginal bleeding during pregnancy, as well as being significantly more likely to report having developed anxiety and depression during pregnancy (Table 5). The increased likelihood of reporting pelvic girdle pain remained after including hypermobility as a covariate and therefore accounting for baseline differences in hypermobility between the groups. The groups did not significantly differ in their likelihood of reporting gestational diabetes, high blood pressure, preeclampsia, eclampsia, infection of the amniotic sac, polyhydramnios, placenta previa, placental abruption or hyperemesis gravidarum.

**Table 5:** Pregnancy conditions

	Non-autistic group	Autistic group	aOR (95% CI)	p-value	p-value (FDR adjusted)
Pelvic girdle pain	145 (28%) *(n = 523)*	144 (35%) *(n = 412)*	1.76 (1.30–2.38)	**< 0.001**	**0.001**
Pelvic girdle pain (with hypermobility as a covariate)			1.57 (1.15–2.15)	**0.01**	**0.01**
Gestational diabetes	40 (8%) *(n = 523)*	45 (11%) *(n = 412)*	1.47 (0.92–2.36)	0.11	0.16
High blood pressure	39 (7%) *(n = 523)*	41 (10%) *(n = 412)*	1.34 (0.83–2.17)	0.23	0.31
Preeclampsia	36 (7%) *(n = 523)*	31 (8%) *(n = 412)*	0.96 (0.56–1.61)	0.87	0.87
Eclampsia	3 (1%) *(n = 523)*	1 (0.24%) *(n = 412)*	0.60 (0.03 - 4.98)	0.66	0.69
Infection of the amniotic sac	4 (1%) *(n = 520)*	1 (0.24%) *(n = 410)*	0.29 (0.01 - 2.25)	0.29	0.38
Polyhydramnios	20 (4%) *(n = 520)*	19 (5%) *(n = 410)*	1.24 (0.63–2.43)	0.53	0.59
Placenta previa	19 (4%) *(n = 520)*	14 (3%) *(n = 410)*	0.85 (0.40–1.75)	0.66	0.69
Placental abruption	12 (2%) *(n = 520)*	8 (2%) *(n = 410)*	0.66 (0.24–1.69)	0.39	0.45
Vaginal bleeding	92 (18%) *(n = 520)*	108 (26%) *(n = 410)*	1.72 (1.24–2.40)	**0.001**	**0.002**
Hyperemesis gravidarum	75 (14%) *(n = 524)*	76 (18%) *(n = 415)*	1.21 (0.83–1.76)	0.32	0.38
Anxiety	72 (14%) *(n = 523)*	157 (38%) *(n = 412)*	3.96 (2.84–5.58)	**< 0.001**	**< 0.001**
Depression	45 (9%) *(n = 523)*	97 (24%) *(n = 412)*	3.21 (2.15–4.86)	**< 0.001**	**< 0.001**

*Note.* Binary logistic regressions performed

[Table 5 here]

## Prenatal Appointments

### Autism Disclosure, Adjustments and Autism Understanding

When asked whether they had disclosed their autism to medical professionals, almost half of autistic respondents indicated that this question was not applicable to them (Table 6). Many indicated in their open-text response that this was because they had not received an autism diagnosis at the time of their most recent pregnancy. Of those who felt that the question was applicable, the majority did not disclose their diagnosis. Participants were marginally more likely to disclose to a doctor (13%) than a midwife (10%) or a sonographer (3%). Participants indicated in an open-text response that their reasons for not disclosing included concern about negative reactions, ‘*I do not think my midwife or doctor would know what that means or what to do with this info. I fear that would make them doubt my feelings and answers and take me less seriousl*y’.

**Table 6:** Autism disclosure, adjustments offered and autism understanding

	N	Yes	No	Don’t know	Not applicable
Disclosed autism to:					
Midwife	411	41 (10%)	169 (41%)	-	201 (49%)
Doctor/GP	413	52 (13%)	169 (41%)	-	192 (46%)
Sonographer	409	14 (3%)	205 (50%)	-	190 (46%)
Disclosed autism to at least one professional	414	59^a^	355^b^	-	-
Adjustments offered:					
Home visits	58	10 (17%)	48 (83%)	-	-
Accompaniment by community midwife to appointments	58	5 (9%)	53 (91%)	-	-
Other	45	12 (46%)	33 (54%)	-	-
	N	Agree	Disagree	Don’t know	Not applicable
Health professionals have had a good understanding of how being autistic affects me:					
Midwife	401	30 (7%)	50 (12%)	46 (11%)	275 (69%)
Doctor/GP	399	33 (8%)	67 (17%)	51 (13%)	248 (62%)
Sonographer	400	19(5%)	46 (11%)	55 (14%)	280 (70%)

^a^ This reports the number of autistic participants who disclosed to at least one of: midwife, doctor/GP, or sonographer

^b^ This reports the number of autistic participants who did not disclose to at least one of: midwife, doctor/GP, or sonographer (this number includes those who felt the question was not applicable)

59 autistic participants disclosed their diagnosis to at least one professional during pregnancy (i.e. they disclosed to at least one of the following: midwife, doctor or sonographer). Those who indicated they had disclosed to at least one professional were asked if any adjustments had been made for them. The majority (83%) reported that they were not offered home visits nor for a community midwife to accompany them to appointments (91%). 12 participants indicated that they were offered another form of adjustment. The open text responses indicated that these adjustments included being able to wait for appointments in a quiet area, having blood tests done at home, longer appointment times, having an advocate and being allocated a temporary social worker.

When asked whether they felt that medical professionals had a good understanding of how autism affected them during pregnancy, the majority indicated that this was not applicable (possibly due to not having disclosed their autism or not having been diagnosed). Those for whom this question was applicable tended to disagree that professionals had a good understanding of how autism affected them.

[Table 6 here]

### Attending Prenatal Appointments

A multivariate binary logistic regression was performed for questions concerning attending prenatal appointments. A model including group as a predictor was not a significantly better fit than the model without group, *X*^2^(3) = 3.53, p = 0.38, indicating that the groups did not significantly differ in their likelihood of attending ultrasound, midwife and doctor appointments as a whole (Table 7).

**Table 7:** Attendance of prenatal appointments

	Non-autistic group				Autistic group			
	N	Yes	No	N/A	N	Yes	No	N/A
Attended all ultrasound appointments	524	514 (98%)	7 (1%)	3 (1%)	417	392 (94%)	18 (4%)	7 (2%)
Attended all midwife appointments	524	457 (87%)	6 (1%)	61 (12%)	416	324 (78%)	21 (5%)	71 (17%)
Attended all doctor/GP appointments	523	465 (89%)	5 (1%)	53 (10%)	414	356 (89%)	18 (4%)	40 (10%)

[Table 7 here]

### Other Aspects of Prenatal Appointments

For the remaining questions concerning prenatal healthcare, a multivariate binary logistic regression was performed. A model including group as a covariate was a better fit than the model without group, *X*^2^(13) = 467.21, p < 0.001. The autistic group were significantly more likely than the non-autistic group to feel overwhelmed by the sensory environment of prenatal appointments (76% vs. 14%; Online Resource 4, Supplementary Table 3).

The autistic group were significantly more likely to report seeing a greater number of midwives throughout their pregnancy than the non-autistic group, yet were more likely to feel that seeing the same midwife at each appointment was important to them (77% vs. 68%). The autistic group were more likely than the non-autistic group to find it stressful when they saw a professional who they were not expecting to see at an appointment (68% vs. 37%) and more likely to agree that being informed of which professional they would see in advance of an appointment would be helpful (86% vs. 59%).

The autistic group were significantly less likely than the non-autistic group to feel that professionals took their questions and concerns seriously (55% compared with 84% of the non-autistic group), less likely to feel comfortable asking questions to professionals (57% vs. 90%), less likely to feel that professionals treated them respectfully (63% vs. 88%), less likely to trust professionals (57% vs. 87%) and more likely to feel negatively judged by professionals (54% vs. 26%).

The autistic group were significantly less likely to have received as much information as they would have liked during prenatal appointments (56% vs. 80%) and were significantly less likely to be satisfied with the way in which information was presented to them during prenatal appointments (61% vs. 85%). When asked in an open-text question to describe in what format they would prefer to receive information, both groups felt they would have benefitted from more written information, and for the autistic group this was sometimes linked to difficulty processing verbal information, ‘*I have auditory processing difficulties so having it written I could have digested it easier*’ (autistic participant).

The autistic group were significantly less likely than the non-autistic group to report that they knew when to seek help with pregnancy concerns (67% vs. 89%). The groups did not significantly differ on whether or not they had someone to advocate for them during prenatal appointments. Among those who reported having an advocate, the autistic group were significantly more likely than the non-autistic group to feel that this was helpful (85% vs. 67%). Similarly, among those who reported not having an advocate, the autistic group were significantly more likely than the non-autistic group to feel that having someone to advocate for them would have been helpful (53% vs. 18%).

The autistic group were significantly less likely than the non-autistic group to report being satisfied with the healthcare they received during pregnancy (70% vs. 91%).

### Antenatal Classes

The groups did not significantly differ on whether they had attended antenatal classes (62% of the non-autistic group and 63% of the autistic group attended; Online Resource 4, Supplementary Table 4). The autistic group were significantly more likely to find it difficult to attend antenatal classes (56% vs. 14%).

For the six questions about difficulties with antenatal classes, a multivariate binary logistic regression was performed. A model including group as a predictor was a better fit than the model without group, *X*^2^(6) = 57.34, p < 0.001. The autistic group were significantly more likely than the non-autistic group to agree that the size of the group at antenatal classes is too large (72% vs. 30%), that antenatal classes are too noisy (64% vs. 19%), that there is too much pressure to socialise at antenatal classes (87% vs. 54%), that information at antenatal classes is presented too quickly (41% vs. 18%) and that the content of antenatal classes can be distressing (31% vs. 15%). The groups did not significantly differ in their tendency to feel that the content of antenatal classes was not useful to them, with the minority of both groups (45% of the autistic group and 31% of the non-autistic group) reporting that classes were not useful.

### Support

For questions concerning support from partners, friends and family, a multivariate binary logistic regression was performed. A model including group as a predictor was a better fit than the model without group, *X*^2^(3) = 106, p < 0.001 (Online Resource 4, Supplementary Table 5). The autistic group were significantly less likely to have received all the support they needed from their partner/spouse (62% vs. 80%), family (50% vs. 77%) and friends (51% vs. 85%). The majority of the autistic group (95%) had not received peer support from other autistic parents. Of those who did receive peer support, 100% of those who responded indicated that they found this support helpful. Of those who did not receive peer support, 59% of those who responded would have found such support helpful.

## Discussion

This is the first in depth quantitative study of the pregnancy experiences of autistic people. The findings indicate lower perceptions of prenatal healthcare as well as atypical physical and sensory experiences during pregnancy among autistic people.

Physical experiences during pregnancy were found to differ among autistic and non-autistic people. Sensory experiences during pregnancy were heightened, and more likely to lead to feeling overwhelmed, among the autistic group. This was the case not only for smell and taste but also for touch, hearing and vision - senses less commonly associated with changes during pregnancy. It is worth noting that the autistic group were also more likely to report a reduction in touch and hearing compared with the non-autistic group, in keeping with prior evidence of both hyper- and hyposensitivity in autistic people (Ben-Sasson et al., 2009). The autistic group were more likely to report interoception and proprioception changes during pregnancy as well as to report difficulties adapting to the physical changes of pregnancy, indicating that adjusting to the somatic changes of pregnancy may be particularly challenging for autistic people. Autistic participants also experienced nausea more frequently than non-autistic participants, which may plausibly be influenced by a greater increase in intensity of smell and taste among this group. Autistic participants frequently experienced shutdowns and meltdowns during pregnancy, with over half experiencing meltdowns and shutdowns at least once a fortnight and the majority reporting a greater intensity of meltdowns and shutdowns during pregnancy. A minority of the non-autistic group also reported meltdowns and shutdowns, though it is unclear whether these experiences are qualitatively similar to those of autistic people. Greater meltdowns and shutdowns during pregnancy may be influenced by the stresses of heightened sensory and other physical experiences in addition to difficulties accessing appropriate healthcare.

Evidence was found of increased rates of pelvic girdle pain and vaginal bleeding among the autistic group. The increased risk of pelvic girdle pain may partially be explained by increased hypermobility among autistic people (Cederlöf et al., 2016), though this is unlikely to provide a full explanation given that the group difference remained significant after controlling for hypermobility. The increased risk may also be due to the fact that autistic people tend to be at greater risk of chronic pain than non-autistic people (Whitney & Shapiro, 2019). An increased risk of vaginal bleeding may in part be due to hormonal factors, given that differences in endocrine system function have been associated with autism (Sarachana et al., 2011). The finding of no increased risk of gestational diabetes fits with the findings of Sundelin et al., (2018) though the finding of no increased risk of preeclampsia is in contrast with the findings of this paper. It may be that null findings concerning pregnancy conditions are due to having a smaller sample size than would typically be expected for epidemiological studies. A post-hoc power analysis for preeclampsia indicated that for the total sample of 935, there was adequate (80%) power to detect an odds ratio of ≥ 1.94, with a two-tailed alpha of 0.05, indicating that null results may have been due to a lack of power. It is also possible that pregnancy conditions may be underdiagnosed among autistic people, given that 89% of participants in the present sample did not know when to seek help with pregnancy concerns. It is important that healthcare professionals are aware that autistic people may face increased risk of pregnancy conditions such as pelvic girdle pain and vaginal bleeding and that autistic patients may benefit from guidance on when to seek help with physical issues during pregnancy.

Greater prenatal anxiety and depression among the autistic group than the non-autistic group fit with findings of an increased prevalence of mental health difficulties among autistic people compared with the general population (Lai et al., 2019) and increased prenatal and postnatal depression among autistic women (Pohl et al., 2020). It may be that the stressors of increased physical issues during pregnancy and lower satisfaction with maternity care may contribute towards greater feelings of anxiety and depression among autistic people during this time.

Autistic participants had lower perceptions of prenatal healthcare than non-autistic participants. In keeping with the findings of Gardner et al., 2016, autistic participants were more likely to feel overwhelmed by sensory experiences during prenatal appointments. This indicates the need to make appointments more accessible for autistic people by reducing sensory stimuli. Replicating previous findings (Pohl et al., 2020), autistic and non-autistic participants were just as likely to attend antenatal classes, though the findings indicate that several aspects of antenatal classes may not be suitable for autistic people and that smaller classes with less pressure to socialise may be more appropriate. The autistic group were more likely to consider continuity of care to be important, yet saw a greater number of midwives during their pregnancy compared with the non-autistic group (possibly due to having also seen specialist midwives). Ensuring continuity of care may be an important adjustment for autistic people, in addition to being kept informed of who will be providing their care.

Echoing prior findings that autistic mothers prefer not to disclose their autism diagnosis to professionals (Gardner et al., 2016; Pohl et al., 2020), participants tended not to disclose to various health professionals during prenatal appointments. Similar to previous findings (Pohl et al., 2020), participants may not have disclosed due to fear of negative attitudes from professionals. Indeed autistic participants were more likely to feel judged by and unable to trust professionals, and less likely to feel treated with respect in appointments. This is in keeping with findings that autistic mothers are more likely to feel misunderstood by professionals (Pohl et al., 2020). Research seeking the perspectives of professionals themselves would be valuable in order to establish what attitudes healthcare professionals hold towards autistic mothers.

Participants were often not offered autism-related adjustments during appointments and did not always feel that professionals had a good understanding of autism. These findings fit with previous findings that people with disabilities commonly feel that reasonable adjustments are not made for them in maternity appointments and that professionals do not have sufficient awareness of disability (Hall et al., 2018). However, it is important to note that some participants may not have received adjustments due to not having received a diagnosis of autism or not having disclosed their diagnosis.

Autistic participants were less satisfied with the way in which information was presented to them in appointments. This fits with prior findings that autistic people experience communication-related barriers to healthcare (Nicolaidis et al., 2015; Raymaker et al., 2017; Rogers et al., 2017) and that autistic mothers are more likely to experience issues communicating with professionals about their child (Pohl et al., 2020). It also builds on findings that people with disabilities are less likely to be spoken to by professionals in a way they could understand (Malouf et al., 2017; Redshaw et al., 2013).

The autistic group were also less satisfied with support from informal sources such as partners, friends and family. Peer support from other autistic parents was desired by most participants, though only 5% had received peer support during pregnancy. The provision of peer support (perhaps in the form of pregnancy peer support groups) may therefore be important for ensuring autistic people’s wellbeing during pregnancy.

### Limitations

The sample may not be representative of all autistic parents. Many of the autistic group did not have a diagnosis of autism or may not yet have been diagnosed at the time of their most recent birth. Parents without a diagnosis may have different experiences to those with a diagnosis, including being treated differently by professionals and receiving fewer adjustments. Further, the survey may not have been accessible all to parents, such as those with an intellectual disability. The experiences of autistic parents were not compared with those of parents with other conditions, including mental health conditions, meaning that it is unclear whether the issues raised are specific to autistic parents or common to disabled parents more broadly. The sample was also predominantly composed of participants of white ethnicity from western countries and may not be representative of other populations. It is also possible that some group differences seen may be influenced by other factors such as differences in socio-economic status and gender identity.

Participants often reported on experiences that occurred several years ago and as such, their recollection may not have always been reliable. In addition, retrospective reports of experiences may not reflect more recent healthcare. Furthermore, participants from a range of different countries are represented and it is therefore not possible to draw conclusions specific to any particular country’s healthcare system. The survey relies on self-report and as such, triangulation using other methods such as medical records and studies seeking the perspectives of professionals are necessary to corroborate findings. The latter would help to establish the level of autism-related knowledge maternity professionals possess and the attitudes they hold.

## Conclusion

This study identifies gaps in prenatal healthcare for autistic people and highlights the need for adjustments to be made. These include the provision of information in a variety of formats, adjustments to the sensory environment of appointments and the presence of an advocate during appointments. Due to difficulties accessing group-based support, the provision of antenatal classes in alternative formats such as one-to-one or online classes may be beneficial for autistic people. Greater autism awareness among healthcare professionals, in addition to greater continuity of care may help to build trust between professionals and their autistic patients. Fear of a lack of understanding from professionals may be a barrier to disclosure of an autism diagnosis and therefore may be a barrier to accessing adjustments and support. Furthermore, greater mental health support for autistic people during pregnancy is essential, given an increased risk of prenatal depression and anxiety.

**Supplementary Information**.

ESM_1: Tables showing correlations between items on the survey.

ESM_2: Tables showing results of statistical analyses for selected findings.

## Data Availability

The anonymised dataset is available on reasonable request from the corresponding author.

## References

[CR1] Allison C, Auyeung B, Baron-Cohen S (2012). Toward brief “Red Flags” for autism screening: The Short Autism Spectrum Quotient and the Short Quantitative Checklist for Autism in toddlers in 1,000 cases and 3,000 controls [corrected]. Journal of the American Academy of Child and Adolescent Psychiatry.

[CR2] Ben-Sasson A, Hen L, Fluss R, Cermak SA, Engel-Yeger B, Gal E (2009). A meta-analysis of sensory modulation symptoms in individuals with autism spectrum disorders. Journal of Autism and Developmental Disorders.

[CR3] Braun V, Clarke V (2006). Using thematic analysis in psychology. Qualitative Research in Psychology.

[CR4] Cage E, Di Monaco J, Newell V (2018). Experiences of Autism Acceptance and Mental Health in Autistic Adults. Journal of Autism and Developmental Disorders.

[CR5] Cederlöf M, Larsson H, Lichtenstein P, Almqvist C, Serlachius E, Ludvigsson JF (2016). Nationwide population-based cohort study of psychiatric disorders in individuals with Ehlers–Danlos syndrome or hypermobility syndrome and their siblings. Bmc Psychiatry.

[CR6] Cohen J (1992). A power primer. Psychological Bulletin.

[CR7] Gardner M, Suplee PD, Bloch J, Lecks K (2016). Exploratory Study of Childbearing Experiences of Women with Asperger Syndrome. Nursing for Women’s Health.

[CR8] Hall J, Hundley V, Collins B, Ireland J (2018). Dignity and respect during pregnancy and childbirth: A survey of the experience of disabled women. BMC Pregnancy and Childbirth.

[CR33] Hampton, S., Allison, C., Baron-Cohen, S., & Holt, R. (2022). Autistic People’s Perinatal Experiences II: A Survey of Childbirth and Postnatal Experiences. *Journal of Autism and Developmental Disorders.*10.1007/s10803-022-05484-4 10.1007/s10803-022-05484-4 PMC10290578 35445371

[CR9] Henderson J, Jomeen J, Redshaw M (2018). Care and self-reported outcomes of care experienced by women with mental health problems in pregnancy: Findings from a national survey. Midwifery.

[CR10] Homeyard CE, Patelarou E (2018). To what extent are midwives adapting antenatal information for pregnant women with intellectual disabilities? A survey of NHS trusts in England. Public Health.

[CR11] Homeyard C, Montgomery E, Chinn D, Patelarou E (2016). Current evidence on antenatal care provision for women with intellectual disabilities: A systematic review. Midwifery.

[CR12] Lai, M. C., & Baron-Cohen, S. (2015). Identifying the lost generation of adults with autism spectrum conditions.The Lancet Psychiatry, *2*(11),1013–1027. 10.1016/S2215-0366(15)00277-1 10.1016/S2215-0366(15)00277-1 26544750

[CR14] Lai MC, Kassee C, Besney R, Bonato S, Hull L, Mandy W, Szatmari P, Ameis SH (2019). Prevalence of co-occurring mental health diagnoses in the autism population: A systematic review and meta-analysis. The Lancet Psychiatry.

[CR15] Lewis, L. F. (2017).A Mixed Methods Study of Barriers to Formal Diagnosis of Autism Spectrum Disorder in Adults.Journal of Autism and Developmental Disorders, *47*(8),2410–2424. 10.1007/s10803-017-3168-3 10.1007/s10803-017-3168-3 28516422

[CR17] Lockwood Estrin, G., Milner, V., Spain, D., Happé, F., & Colvert, E. (2021). Barriers to Autism Spectrum Disorder Diagnosis for Young Women and Girls: A Systematic Review.Review Journal of Autism and Developmental Disorders, *8*(4),454–470. 10.1007/s40489-020-00225-8 10.1007/s40489-020-00225-8 PMC8604819 34868805

[CR19] Malouf R, Henderson J, Redshaw M (2017). Access and quality of maternity care for disabled women during pregnancy, birth and the postnatal period in England: Data from a national survey. British Medical Journal Open.

[CR20] Nicolaidis C, Raymaker DM, Ashkenazy E, McDonald KE, Dern S, Baggs AE, Kapp SK, Weiner M, Boisclair WC (2015). “Respect the way I need to communicate with you”: Healthcare experiences of adults on the autism spectrum. Autism.

[CR21] Noonan M, Jomeen J, Galvin R, Doody O (2018). Survey of midwives’ perinatal mental health knowledge, confidence, attitudes and learning needs. Women and Birth: Journal of the Australian College of Midwives.

[CR22] Pohl AL, Crockford SK, Blakemore M, Allison C, Baron-Cohen S (2020). A comparative study of autistic and non-autistic women’s experience of motherhood. Molecular Autism.

[CR23] Raymaker DM, McDonald KE, Ashkenazy E, Gerrity M, Baggs AM, Kripke C, Hourston S, Nicolaidis C (2017). Barriers to healthcare: Instrument development and comparison between autistic adults and adults with and without other disabilities. Autism.

[CR24] Redshaw M, Malouf R, Gao H, Gray R (2013). Women with disability: The experience of maternity care during pregnancy, labour and birth and the postnatal period. BMC Pregnancy and Childbirth.

[CR25] Rogers C, Lepherd L, Ganguly R, Jacob-Rogers S (2017). Perinatal issues for women with high functioning autism spectrum disorder. Women and Birth.

[CR26] Sarachana T, Xu M, Wu RC, Hu VW (2011). Sex Hormones in Autism: Androgens and Estrogens Differentially and Reciprocally Regulate RORA, a Novel Candidate Gene for Autism. Plos One.

[CR27] Sundelin HE, Stephansson O, Hultman CM, Ludvigsson JF (2018). Pregnancy outcomes in women with autism: A nationwide population-based cohort study. Clinical Epidemiology.

[CR28] Talcer, M. C., Duffy, O., & Pedlow, K. (2021).A Qualitative Exploration into the Sensory Experiences of Autistic Mothers.Journal of Autism and Developmental Disorders. 10.1007/s10803-021-05188-1 10.1007/s10803-021-05188-1 PMC9944021 34251566

[CR30] Whitney DG, Shapiro DN (2019). National Prevalence of Pain Among Children and Adolescents With Autism Spectrum Disorders. JAMA Pediatrics.

[CR31] Whitney, D., & Stansfield, A. J. (2019). Should we be accepting self-referrals for Autism assessments? *Advances in Autism, 6*(2), 121–127.

